# The role of accommodation environments in student mental health and wellbeing

**DOI:** 10.1186/s12889-021-10602-5

**Published:** 2021-03-23

**Authors:** Joanne D. Worsley, Paula Harrison, Rhiannon Corcoran

**Affiliations:** 1 Department of Primary Care and Mental Health, University of Liverpool, Liverpool, UK; 2 Student Administration and Support, University of Liverpool, Liverpool, UK

**Keywords:** Transition, Attachment, Mental health, Wellbeing, Relational spaces, Loneliness, First year students

## Abstract

**Background:**

Due to the increasing concern over student mental health and wellbeing, attention has turned to the matter of creating environments, communities, and institutions which enable students to flourish.

**Methods:**

To explore the role of accommodation environments in first year student mental health and wellbeing, eight focus groups were conducted in two universities in North West England and analysed using thematic analysis.

**Results:**

Three overarching themes were identified: ‘*The betwixt space*’; ‘*Accommodations as vessels to cultivate friendships and communities*’; and ‘*The importance of accommodation-based pastoral staff*’. As attachment to place and relationships with significant others are disrupted by the transition, this leaves young people vulnerable whilst they go through a process to re-attach to new people and a new environment, and loneliness and social isolation were keenly felt during this period. Physical attributes of place that prevent social cohesion further exacerbated feelings of loneliness.

**Conclusions:**

As it is common for students to withdraw physically and psychologically when they do not form friendships within their flat, accommodation-based pastoral staff have an important role to fulfil. Although these findings demonstrate the importance of the human element within accommodation, designing places that facilitate community, a sense of we-ness, and belonging is crucial.

**Supplementary Information:**

The online version contains supplementary material available at 10.1186/s12889-021-10602-5.

## Background

Starting university is a major period of transition requiring young people to adapt to new environments and social situations [1]. According to a recent report by Unite Students [2], living in shared halls of residence is the most popular choice of accommodation for university applicants in the United Kingdom (UK). However, there are a number of challenges associated with moving from home into student accommodation such as living with strangers, developing independence, and managing domestic duties and needs [3]. The transition between one place (e.g., the home environment) and another place (e.g., the university environment) has been conceptualised as a *betwixt space* [4], and it is common for mental health problems to arise whilst students are acclimatising to their new environment. According to a UK cohort study, levels of psychological distress increase on entering university and do not return to pre-registration levels throughout the degree course [5]. Due to the increasing concern over student mental health and wellbeing, attention has turned to the matter of creating environments, communities, and institutions which enable students to flourish. The Universities UK (UUK) ‘Step Change’ initiative for improving student mental health and wellbeing emphasises the importance of a holistic ‘whole institution approach’ [6]. As student accommodation is where many students spend most of their time [7], an important aspect of the student experience is the physical environment and living space. Despite UUK emphasising a ‘whole university approach’ to mental health and wellbeing, there is limited research exploring the impact of accommodation environments on student mental health and wellbeing.

The World Health Organisation (WHO) reports that people in Europe spend 90% of their time indoors [8]. It is therefore not surprising that a link between where a person lives and their mental health exists [9]. For university students, this is heightened by the uniqueness of their living environment as many relocate from their family home to live alongside hundreds of other young people who they have never met before [7]. Recognising this link, Simon Fraser University in Canada has published a number of briefings around designing physical spaces for wellbeing which align with best practice criteria for built environments including the WELL Building Standard [10] and the Whole Building Design Guide [11], and acknowledge the importance of natural light, providing opportunities for social interaction, connecting buildings to nature, and enabling control over furniture choice. Designing physical spaces for wellbeing is of importance given that spaces are not inanimate background on which life ‘happens’ and objects ‘are’. Instead spaces condition the way individuals live and interact with others [12]. As relational spaces can afford relational wellbeing, defined as feeling good and functioning well together [13], the design of student accommodation is important.

Indeed, the internal design of student accommodation has important effects on interpersonal relationships and wellbeing. For example, in research conducted in the UK, Easterbrook and Vignoles [14] found that the availability of shared communal areas increased the frequency of coincidental meetings between students in shared accommodation, which in turn facilitated greater interpersonal bonds and enhanced feelings of wellbeing. Thus, physical proximity and face-to-face meetings act as a catalyst for friendship formation. The importance of architecture in shaping first-year student engagement and experiences has been highlighted by Brown, Volk, and Spratto [15] who found that US students living in social corridor residence halls, characterised by both communal spaces for interaction and limited barriers to privacy, reported higher academic outcomes than those living in isolating apartment residence halls, characterised by successive locking doors and minimal communal space. While this insight is invaluable, it is important to note that this literature is quantitative and that a more detailed insight on accommodation spaces as a wider determinant of wellbeing, such as that captured by qualitative methods, is desirable.

According to a recent report by Unite Students [2], 88% of young people in the UK who were planning to move away from their family home rated living with other people who they like as more important than the specification of their accommodation. For many young people, their primary support system will shift away from their family and high-school friends, presenting a unique opportunity to foster new relationships [16]. Due to the security new relationships afford, students recognise the importance of forming new friendships quickly during the transition period [17]. New friendships formed when students begin university are important in terms of helping adjustment to a new social environment [18]. Students who are well integrated with others in their accommodation are also less likely to consider dropping out of university [19].

Forming new friendships at university can be difficult, however. The first year of university can be a particularly lonely time for UK undergraduate students [20], and the most frequently identified period that students will likely experience loneliness is during the first 3 months of university [21]. Jopling and Valtorta [21] found that, on average, UK students were spending 4.2 h a day alone in their room, and 35% of students who reported spending longer than 4 hours alone also reported not having friends to spend time with. Many UK students expect to interact more regularly with others at university more than school, and often students are disappointed when this does not happen [22]. According to Neves and Hillman [23], a quarter of UK students who rated their university experience as worse than expected claimed that this was due to limited interaction with other students.

Given the existing research in this area is largely quantitative, the perspectives of students themselves are currently under-represented in this literature. Accordingly, there is a need for qualitative research that can provide in-depth data on the impact of accommodation environments on student mental health and wellbeing. In particular, little is known about student perspectives on the stressors in accommodation environments, and the steps that accommodation teams could take to better support students’ mental health and wellbeing. We aim to explore the unique transition into university with an emphasis on the role of accommodation environments in student mental health and wellbeing.

## Methods

### Ethical approval

Ethical approval was received from the Health and Life Sciences Research Ethics Committee (5762). All methods were carried out in accordance with relevant guidelines. Informed consent was obtained from all participants.

### Participants

Eight focus groups were conducted across two universities in northern England. A total of 38 students (33 females and 5 males) participated in the focus groups: 19 students living in university-owned accommodation (UOA) whilst studying at a Russell Group university and 19 students living in privately owned accommodation (POA) whilst studying at a post 92 institution. Russell Group universities are regarded as research-orientated universities, whereas post-1992 universities are mainly teaching orientated institutions, such as polytechnics, that were awarded a university charter in 1992. Participants were recruited to take part in the study via bulk emails and flyers. Consistent with the population demographics of first-year undergraduates studying at the two universities, the majority of participants were White British and aged between 18 and 19 years. Although no formal data was collected about courses being studied, during the focus groups it emerged that student participants were studying a range of courses across different faculties including Psychology, Physiotherapy and Fashion Communications.

### Focus groups

Focus groups followed a semi-structured set of questions. The topic guide comprised four sections focusing on the transition period, student accommodation, expectations of university life, and friendships (see supplementary file 1). All of the focus groups were conducted by the first author in November and December 2019. As the researcher had no prior relationship with the participants, focus groups were conducted in students’ halls of residence so the environment was familiar to them, whilst ensuring that there was an appropriate member of staff to draw on if required. Given our focus on relational spaces, focus groups were deemed to be an appropriate method of data collection. As the researcher adopted a peripheral role by acting as a ‘facilitator’ to encourage an interactive discussion, focus groups engendered collegiality whilst enabling students to feel comfortable discussing their views alongside peers or similar others who had shared experiences.

### Analysis

Focus group data were thematically analysed by the first author [24]. Thematic analysis is a commonly used method for describing, analysing and reporting themes and patterns in data [24]. The procedure of thematic analysis outlined by Braun and Clarke [24] was adhered to. Initial and repeated viewing of the transcripts was undertaken, considering both contextual and reflective notes. Although there were some pre-determined areas the researcher wanted to explore, line by line coding derived from a largely inductive approach ensured that data were not overlooked. Research meetings were held throughout the coding process to examine emerging impressions of the data, and to discuss and iteratively refine new codes. The initial themes and subthemes captured by coding were refined during discussions with another member of the research team to produce the final themes. This process ensured that the final themes were not just the personal interpretation of one team member.

## Results

Three overarching themes were identified from the data: ‘*The betwixt space*’, ‘*Accommodations as vessels to cultivate friendships and communities*’, and ‘*The importance of accommodation-based pastoral staff*’. A number of subthemes were identified in relation to each overarching theme (see Table 1).

**Table 1:** Overarching themes and subthemes

Themes	Subthemes
The betwixt space	Adjustment and re-attachment
	Loneliness and social isolation
	Expectations
	Self-development with others
Accommodations as vessels to cultivate friendships and communities	Throwing people together: My kind of people/not my kind of people
	Developing a sense of community
	Physical design of spaces
The importance of accommodation-based pastoral staff	

### Theme 1: The betwixt space

#### Adjustment and re-attachment

When young people move from home to a new university environment, they invariably go through a process of adjustment. During this process, students have to start thinking about many factors and aspects of life that they had not previously considered:

*“Since getting here it has been a massive adjustment. I didn’t quite realise the amount of independence you would have and that’s not necessarily positive independence. Also the fact that there are so many things that I’ve got to think about that aren’t thought about for me anymore and so that’s the biggest adjustment. It’s harder than I realised it would be. Suddenly realising that I’ve got no gym kit left and having to wear old stuff because I can’t afford washing”* (FG2 UOA p5)*.*

*“There’s just little things like you don’t have to worry about food in the house. Well I didn’t because I lived at home and my mum would sort that and I didn’t have to think about food. Just little things like that and food. I don’t buy fruit for myself. I’m used to my mum buying it and telling me to eat it and then I realised I’m tired all the time and my diet is absolutely awful. It’s just things like that and I miss having that comfort”* (FG1 UOA p2).

As a young person’s sense of self is closely linked to their environment and relationships, leaving their home environment can affect their wellbeing. Indeed, many students found this period unsettling as they missed their home environment, especially their family members and familiar routines:

*“I really miss my family so it’s been really hard for me. My mum and my sister are my best friends so I’ve found it really hard being away from my family”* (FG3 POA p4).

*“I missed the structure and the normality of home and the familiarity of it”* (FG1 UOA p2).

As young people’s primary support network becomes remote, a unique opportunity presents itself to develop new friendships and establish a new support network:

*“I suddenly realised two weeks in I have zero support network here. I have my mum on Facetime. I have my boyfriend on Facetime and maybe like a lecturer but other than that you have no support network here so you have to build it yourself. I had a bit of a nervous breakdown two weeks in when I just realised I’ve got no one right there like I would at home. At home, I’ve got my mum and I can just go to my mum and have a hug but I have to Facetime her and that’s not really the same*. *So that is what I realised you just don’t make your best mates as quickly as everyone says you do”* (FG2 UOA p5).

*“I think like with mental health, my mum was quite worried about me coming so she would ring me but I was like no I need to go and find other people who I can talk to and I think if I didn’t have those people here now I would’ve really struggled”* (FG4 UOA p3).

Many students felt under pressure to form new friendships as quickly as possible. During the first few weeks, students found themselves evaluating other people’s attributes and values in order to assess whether they would be suitable:

*“There’s a lot of pressure when you’re talking to people as you are like ‘are we going to be best friends?”* (FG2 UOA p4).

*“I think you have to take a step back and look at people and think ok they are similar to me or similar enough and we actually complement each other rather than being sucked into that I’ve got to make as many friends as possible and they’re really popular, they are really cool”* (FG2 UOA p5).

Pressure to attend all of the events during Fresher’s week in order to cultivate a wider friendship network was also keenly felt, and many students viewed this in an instrumental way as a search with a purpose:

*“I might meet this one mystic best friend at this Fresher’s event. I must go*!” (FG2 UOA p4).

*“It’s weird because it's like if I don’t go to this one event then I’m not going to have friends forever”* (FG2 UOA p1).

*“I went out pretty much every night during fresher’s and I hate going clubbing but I forced myself to do it so that I fit in and we went into a nightclub and I had the biggest panic attack of my life in front of them because I had forced myself to try and fit in”* (FG4 UOA p1).

As well as forming new attachments to new people, students also have to attach to a new environment that they can think of as, at least, a temporary home. During this period, students valued having their own private space in order to retreat:

*“I also like the rooms that we still have the private space”* (FG1 POA p1).

*“Having your own space. Like if we had to share rooms or the rooms are smaller than they are I’d have a meltdown. I couldn’t be around people for that long”* (FG2 POA p4).

For many students, their private space was viewed as a place of comfort, individuality, and personal identity. Bedrooms seemed to play an important role as a means of self-expression:

*“I’ve made my room how I want to be, I’ve made it really really comfy so my bed is gorgeous and having photos around my room of my friends and home and making sure my little space was comfortable for me so even if I was having a bad time outside it, I could go back in and just feel way more relaxed”* (FG4 UOA P3).

*“I think the room thing, making it your own, and making it somewhere so that when you do come back, you express yourself through your room as cliché as that sounds, it is just something to look forward to when you come back”* (FG4 UOA P4).

The objects that new students brought from home such as quilts, fairy lights, pictures, and posters were important in providing continuity in students’ lives and worked by connecting the university to home:

*“I tried to make the way I live, I kind of tried to make it a little bit like the things I enjoyed at home. I tried to set them up here so I really like fairy lights and stuff like that so I made sure my room had fairy lights in it so that it was all cosy on an evening. That helped me”* (FG3 POA p3)

*“I’ve got 160 posters over my walls and photos and everything so I’ve been ordering them in every month. My walls are literally plastered in them and it just makes you feel a lot more at home”* (FG3 POA p1).

*“All of my nice bedding is in university so that’s more like my room now”* (FG4 UOA p4).

These objects help students to settle in, and some young people start to refer to their student accommodation as ‘home’:

*“When I’ve been to visit people and I come back it is like coming back home and the fact that our bedrooms are decorated in a way that’s like home. I’ve just brought all the stuff from home”* (FG1 UOA p1).

*“I’ve been home twice and both times I’ve not liked it as I’ve felt like my room was a hotel room. Like none of my blankets were there, my mum rearranged it and I was like this feels weird. I felt like a guest in my own house”* (FG4 UOA p3).

Nevertheless, other students felt suspended in a transient space between home and university, and this notion of liminality may leave students feeling as though they do not belong:

*“I can’t call here ‘home’ and I can’t call home ‘home’ anymore. I’m a lost soul somewhere in the middle”* (FG4 POA p1).

This highlights the uncomfortable nature of the transition period as students often spend time in a betwixt space whilst going through a process of re-attachment to new people and a new environment. During this period, it is common for students to experience homesickness:

*“I’ve moved quite a lot so I didn’t expect to have homesickness and then one of the guys was feeling odd and anxious and I had a chat and it turns out he was just really homesick and he was thinking about quitting university so it just came out of nowhere for him. Then mine started manifesting the third week in. I was feeling a bit anxious and that I was missing my friends”* (FG3 UOA p5).

As some students often feel as though there is something wrong with them when they struggle with the transition, talking to flatmates helps to normalise these feelings as they learn that others are also experiencing similar feelings:

*“Everyone seems so well adjusted and then someone said to me that you seem like you’re handling it so well and I was like do I? I didn’t realise I was. I think everyone doesn’t realise how much other people are struggling actually”* (FG2 UOA p5).

*“That for me was a month when I felt really isolated because there are the twins, who I am now friends with now I’ve got to know them but they struggled as well. So we were all struggling but we didn’t know each other enough to say like we need the help that we can give each other”* (FG4 POA p4).

#### Loneliness and social isolation

As many students move to a new city or country to begin their university journey, they often experience loneliness during the first few months. Loneliness and social isolation were dominant themes across all focus groups:

*“You’re very alone. It is just you in a completely new environment with strangers and completely new settings”* (FG1 UOA P2).

*“I just found it was really lonely like when you get here because having a flat with six girls but like no one comes out their rooms and stuff and you do get on with them but it’s different from being at home with your family. It got really lonely when I was here”* (FG2 POA p3).

*“I think it can be quite isolating as well because you move away from your friends and family”* (FG2 POA p1).

Some students reported feeling too scared to use their own kitchen, highlighting that the design of accommodation environments may not cater for all individuals. In fact, it may, inadvertently, foster loneliness:

*“I think for a lot of people it does really scare them and they can shut themselves away at uni because you have got your own room and you can just end up in there because you are too nervous. I think for a lot of people, like I know in the first week there were times when I just sat in my room because I was too nervous to go and cook”* (FG4 UOA p3).

*“I felt I can’t go out into the kitchen. I feel so nervous because I just felt in my head that they had all made this friendship group and I’m *n*o longer in it … I remember standing at my door going to open it and I just couldn’t so I just went back into my chair”* (FG4 UOA p4).

“*I’ve been too scared to go to my kitchen and I’ve literally cried about going to the kitchen because of how anxious it makes me*” (FG4 UOA p1).

Other students highlighted that the kitchen within their flat was not very homely, and this prevented them from socialising in this space:

*“I don’t think that many people tend to chill in the kitchen quite a lot because it’s not a homely environment so when you’re in your own room quite a lot on your own, it can get quite lonely and that can affect people’s mental health because you can start to feel kind of depressed”* (FG1 POA p2).

#### Expectations

Students’ experiences of university life often did not match their preconceived expectations:

*“It just wasn’t what I was expecting at all because my older brother went to uni and he loved it and said it was the best years of his life and he was really jealous that I was going. Then I got here and I was like I just want to be at home because I was just so lonely”* (FG4 POA p3).

*“Everyone told me that going to uni, you’re going to love it. It’s going to be the best years of your life. In the first few weeks, it is definitely more so I can understand it now that I’ve settled and found my friends and got more comfortable and things like that but in the first few weeks I found it really tough because I was like why am I not having the bestest time in the world when I’ve been told I should be. So maybe just an awareness that you know that it’s going to be tough and that’s ok. Everyone finds it tough but it will get better rather than everyone finds it great from the get go”* (FG1 UOA p2).

Many students did not form close relationships with others as quickly as they were expecting to, and a mismatch between the quantity and quality of relationships that students had and those that they expected to have enhanced feelings of loneliness:

*“I think for me it was just the added social media as everyone loves to make out that uni is an incredible place and everyone is having the time of their lives and everyone loves their flats and they are one big family and you are like why is that not like that for me? Should I not have my best friends at this point? Then you have to remember it has only been a week. Stop being so dramatic”* (FG1 UOA p2).

#### Self-development with others

As many students will be experiencing independence for the first time when they move from home to a new university environment, they often arrive without well-developed life skills:

*“I was ringing my mum up in the supermarket. In the first week I had nothing and I didn’t know what to buy or make”* (FG2 POA p3).

*“I think some people are very dependent on their parents maybe. I know some people who couldn’t cook for themselves, some people couldn’t do laundry”* (FG2 UOA p2).

*“Some people in my flat hadn’t made a bed, hadn’t washed up”* (FG1 UOA p1).

As students live alongside other students from different backgrounds, countries, cultures, and religions, they valued the opportunity to learn from others and broaden their horizons:

*“It’s a mix of culture as a lot of them are from different places so I get to learn a lot of new things, culturally”* (FG3 UOA p4).

*“It’s nice having a bit of a culture change because she’s from Myanmar and she told me a bit about it and told me some of the food that they eat and she’s cooked stuff and I tried it so that’s really cool”* (FG3 UOA p5).

However, not all students valued the opportunity to expand their horizons, and many would have preferred to live with similar others:

*“Being an international student, I wish they put international students together let’s say so that I lived with at least one more international person who goes through the same things as me”* (FG1 POA p1).

*“Three didn’t want to drink for religious reasons and some went out all of the time. It was hard for us to all do things together so that was where kind of the issues started”* (FG3 UOA p3).

The new university environment presents the opportunity to engage in a live and conscious experience of self-development as it enables young people to unmask, build, or redefine their identity, and some young people felt comfortable testing different ‘selves’ in this new environment as no one was watching or judging them:

*“I think at home, you have your friends. People know of you and know you as this type of person. But I think actually coming to university is good because you don’t know anyone, you can be who you want to be, you can try new things, you can make friends with different people. Because it is so big you could be one thing one week and another thing next week and no one would realise because no one is watching you”* (FG3 UOA p2).

This live experience of self-development was described by one student as being ‘on stage’ whilst learning how to interact with others and navigating the new social environment:

*“We all come from homes living with four, five other people maybe and then to living somewhere with 700 other people so close and especially the inwards facing flats. It feels very. I wouldn’t say prison-ish but it feels a little bit claustrophobic and then having so many people around, inside your room you feel a bit caged in but outside your room you feel like you’re on stage especially if you haven’t lived with a lot of people. As soon as you go out your door, it’s like I’ve got to put on a face and you feel like you’ve got to act a particular way around other people and you can't act yourself, especially initially”* (FG3 UOA p5).

*“At home you can walk around and not smile at anyone or not talk to anyone because it’s your family. But in the first week of uni, I was like I need to constantly ask how people are, how their day has been, be really smiley, I can’t be moody around these new people so I think that can put a lot of pressure on people when they first get here, to constantly be smiling. It’s a lot of effort”* (FG4 UOA p3).

### Theme 2: Accommodations as vessels to cultivate friendships and communities

#### Throwing people together: my kind of people/not my kind of people

Some accommodation providers allocate young people to a flat in a random or first come first served list-like fashion. One student referred to this process as being ‘thrown’ together:

*“It is strange just being thrown in with four or five other people altogether when you have never met them before. It is such a drastic change from being at home”* (FG1 POA p4).

In some circumstances, random allocation works and several students described themselves as ‘lucky’ if they formed strong friendships with their flatmates:

*“My flat all got on straight away. I think we got quite lucky”* (FG1 UOA p4).

*“I do feel very lucky to be put with a group of girls who are very nice and we have got on really well. Some of my friends are at [name of a different university] and they said I am very lucky that I have a good group of flatmates and they just don’t really socialise because they don’t click with their flatmates and they had no say in that”* (FG4 UOA p4).

*“I know I’ve really lucked out because when I told my friends at home they were like you’re so lucky, you’ve lucked out loads with that because you are just a random group of people put together and the chances of you all getting on are very slim”* (FG4 UOA p4).

There are many advantages of having a friend nearby to talk over the stresses and challenges of student life. Perhaps simply listening when students are experiencing problems is the most important way in which new friends can help. Students highlighted the importance of living with others whom they feel able to confide in or talk to about their problems, providing the social support that is so crucial during the transition period:

*“In my flat we all became, there are five girls and three boys, and us five girls have become really close in the fact that we can open up and stuff. So in the first week when I did end up crying, I could talk to them about what went on and I think if I hadn’t had that, like people I could talk to and I didn’t surround myself by people who I can talk to, I don’t know what would have happened”* (FG4, UOA P3).

*“Although I’ve only known you seven weeks I can just go and talk to you if I’m really upset, or even if you don’t want to talk you can just go for a hug. Sometimes just a bit sad and being in your room moping isn’t going to make you feel better”* (FG1 UOA p2).

On the other hand, however, some students struggle to form close interpersonal bonds with their flatmates:

*“I like the building if that makes sense but the people in my flat are not my kind of people so if I could choose to live with people, it wouldn’t be them. It’s not necessarily that they are not nice people, there has been some friction but they are not nasty people per se”* (FG4 POA p4).

*“Within the first week I found it really difficult because I didn’t really get on with my flat. They are nice enough people, there is nothing wrong with them, they are lovely people but they are just not my kind of person”* (FG1 UOA P2).

Those who experience difficulties forming positive relationships with their flatmates often internalise this, and subsequently feel disappointed or as though there is something wrong with them:

*“Seeing the boys in my flat all gelled really well and I thought why can they do that and I can’t? It was like one of the girls on my course is best friends with her whole flat and I just thought what am I doing wrong? Am I doing something wrong? Am I not being friendly enough?”* (FG4 POA p4).

*“I think when universities advertise it to you it is like ‘you are going to be great friends with your flatmates’. Then you start questioning what’s wrong with me?”* (FG4 UOA, p4).

Students who did not gel with their flatmates often found themselves in confrontational situations or feeling alone:

*“If you are not gelling with your flat then you are on your own and I have really bad OCD and they don’t understand that if you leave pots on the side it’s going to be used and if you leave it dirty it really stresses me out and I don’t think they are considerate of other people, I think that’s what they are lacking and they don’t understand that everyone has got their own issues”* (FG4 POA p1).

*“It was difficult for me because I moved into a flat with 8 other girls so we were a large flat to start off with and due to the majority of us not getting along we actually all spilt up. There were 4 of us who got along and the other 4 got along but as 8 girls together we didn’t get along. There was a lot of arguing, there was a lot of tense, there was a lot of cattiness just because there were a lot of girls so I moved. So it would be over small things such as the fridge, the freezer, leaving dirty dishes so a whole month of that and I just had enough”* (FG3 UOA p3).

Living with incompatible others and experiencing difficulties within flats can have a negative impact on students’ mental health and wellbeing:

*“I have anxiety and depression and living in a flat where I don’t get on with people and I can’t go in the kitchen and stuff it’s getting me down so I can’t sleep until like 4 o’clock in the morning and then I wake up at 3 o’clock in the afternoon and I have no time and then I don’t go to uni and then I feel bad about it so it just gets me down so it’s affecting my mental health”* (FG4 POA p5).

*“I think for me I’d drop out because of what’s happening in the flat and I’m not getting any sleep and I’m already poorly and they’re making me more ill and I just don’t feel like I have the time to do my work because I’m sleeping and I need my sleep”* (FG4 POA p1).

Living with a large number of students caused some young people distress, and often they wished that they lived with a smaller number of students:

*“If I had maybe 5 girls or 4 and then me as the fifth one that would have been easier but when you go in and there are 8 other girls that you are living with and everyone’s got different cultures and everyone’s been brought up differently. So some people will wash a dish straight away whereas others will leave it until they get back. So if you've got that thing were you want the dishes to be washed straight away and another person doesn’t, it then becomes that’s annoying me. If I try to deal with it and say look can you please wash it, and they don't like it, it then starts an argument”* (FG3 UOA p3).

Other students reflected on the number of students within flats, concluding that living with a smaller number of others would present fewer difficulties. The majority of students agreed that flats should be family size:

*“I think it’s better to have a smaller group and just get on with them all and have it be close than have a bigger number”* (FG3 POA p3).

*“This is the problem I see with you, you’re living in groups of 10. That’s why I see it is a bit easier when I’m just living with three other guys … We seem to get on so well and I think because it’s a smaller group as we have gelled a lot easier. There are not loads of different other people from like different backgrounds”* (FG4 POA p2).

With regard to allocation, students would prefer to live with similar others, and many suggested that accommodation teams should launch a personality survey in order to match students with similar others:

*“Some people love their flats whereas some people don’t get along so if you had an extensive bit on the application form to say personality. It might be a little bit easier for some people”* (FG3 UOA p3).

*“I think we should do a quiz before we move in so there’s at least someone who’s got the same interests so you can gel well with at least one person in your flat”* (FG4 POA p1).

#### Developing a sense of community

Some students expressed concerns about the lack of a community feeling within their accommodation:

*“The only thing I would improve is that sense of community like doing things with your floor or block should be done and I know it is all down to us but maybe if it was encouraged more so F block do this and that”* (FG4 UOA p2).

*“As much as we are adults it would be nicer to have more support in getting to know people and stuff like making it feel more of a community because I do feel as though we have just been left to our own devices”* (FG1 POA p4).

As many students highlighted that the only people they know within their accommodation are those living in their flat, formal activities in the social and communal areas arranged by accommodation teams could support the creation of a community feeling:

*“Maybe if there are groups of people who like the same things maybe try and get them together and do a themed night and then you might get people who didn’t know they would get on well and then they can meet each other. I’d just try and make smaller groups because as I’ve said there are too many people for everyone to be friends with everyone but if you kind of get like little groups of different themes or different interests and sort it that way then you might get more of a community”* (FG3 POA p4).

*“If they had things here and they advertised it, like oh this and that is on TV, pool tournament, or pyjama party or just different ideas, I’m sure people would go. It’s just there’s nothing, well the pool tables are there, the TVs are there but no one wants to leave their room and venture downstairs”* (FG4 UOA p2).

*“Even things like Twister and drinks or something like that would be funny or even pizza, Twister and drinks”* (FG3 POA p1).

It was also suggested that accommodation providers could also provide outdoor facilities such as football nets or basketball hoops to encourage students to socialise together in outdoor spaces:

*“I think something that would be kind of cool to see is like we have quite a lot of space at the top so if they set up football goals and stuff we could have a wee football day in the Spring just to get everyone back from Christmas or basketball or something like that. It would be funny to get everyone chatting and stuff”* (FG3 POA p1).

Students also valued the opportunity to talk openly during the focus groups, and would welcome future opportunities to meet similar others in a similar setting:

*“Something like this (referring to the focus group) is really good just a few people in a room getting everything off their chest and talking about things that are important to them”* (FG4 POA p2).

*“I think something like this would be good, I was literally going to say that. Just little meetings and you can just sign up and be like 5 people and you can just go and speak and it can be different every week”* (FG4 POA p3).

#### Physical design of spaces

The design of accommodation spaces can have a really important impact:

*“I think the actual accommodation is really modern and light and it makes me feel happier because I was thinking that a couple of my friends were in accommodation that was dingy and it would make you feel a bit depressed”* (FG2 UOA p1)*.*

When asked what makes a good accommodation, many students highlighted the importance of social and communal spaces within buildings:

*“I like the fact that it’s got this giant common room to just chill in especially during Fresher’s this was like the best thing to have because that’s where you meet everyone during Fresher’s week and then you find your own cliques and everything. I like it because it’s comfortable”* (FG3 POA p4).

*“I think it’s so good the way they have focused on all of the communal spaces because you can be in your room and you don’t have to think I have to spend money to get out the flat. You can just come down and stick a wash on and play table tennis for a bit. That’s really helpful”* (FG4 POA p3).

Students highlighted aspects of the communal and social areas such as natural light, plants, and bright colours as important design features:

*“It’s really bright and the lights are nice, like that purple light, and the green stuff, you know the bits of tree and the plants it just makes it homely and it’s just nice. I just feel at home”* (FG4 POA p4).

Some students, however, suggested ways to improve the social and communal spaces. As these areas should be comfortable spaces for young people, students suggested implementing a number of small changes such as temperature adjustments and the provision of comfier seats:

*“It feels really formal as well like the way the chairs are set out. I think if it was bean bags or comfier chairs then you would just feel more relaxed whereas it just feels like a meeting if you come in here”* (FG2 POA p1).

*“For me I’d say make it more of a comfortable space because I feel like it is quite cold I think and the reason I’d stay in my flat rather than come down here because I want to be warm and cosy whilst I’m revising or just relaxing. I always think if I came and sat here it’s quite hard like I couldn’t relax and settle in”* (FG3 POA p3).

In addition, a number of specific features that encourage or discourage students to use the communal areas within flats were also highlighted:

*“Our sofa is really comfortable and if it was like plastic material I don’t think I would sit on it as much. If that sofa wasn’t there I don’t think I would sit in the kitchen as much”* (FG3 POA p3).

*“Bigger Sofas. There is only space for four but there are six of us. So its either two people have to sit on the stools which aren’t the most comfortable things or everyone has to try and squish onto a tiny sofa”* (FG2 POA p2).

Other students highlighted that the layout of their kitchen does not facilitate communal dining:

*“Even in our kitchen it’s just a couch and our breakfast bar is against the wall so there is literally two chairs at a table staring at a wall so we don’t even have space to have our tea together or anything. Even just having a table with four chairs around it so if we were on placement we could get up and have a cuppa together or come home and have our tea together. I just feel like with our flat we haven't done that because we can’t”* (FG1 POA p4).

*“The kitchens are nice but our table was ridiculous. We had to buy a new one. We only had a breakfast bar facing a wall”* (FG4 UOA p2).

Although accommodation providers refer to the arrangement of rooms as ‘flats’, some students do not feel ‘at home’ in their living space, and one student likened her experience of living in halls not as living in a flat but more like staying in a hostel on a school trip:

*“I feel like I’m just in a hostel for a few weeks on a school trip that’s what I compare it to because its not very homely”* (FG1 POA p4).

Students also highlighted physical attributes of place that prevent social cohesion. For example, one student highlighted the importance of arranging rooms in clusters rather than linear tranches whereas others highlighted that the internal design of shared accommodation may influence student interactions:

*“I wouldn’t want to live in a flat of 16 in [name of accommodation] because it’s just a really long corridor so if you live like half way you have no reason to walk up the opposite direction to the kitchen and I’d feel really awkward just knocking on people’s doors being like ‘hi’”* (FG2 UOA p5).

*“Also like [name of accommodation] when you get out of the lift, you have maybe two flats on one floor so someone in that flat will only ever see their flatmates”* (FG2 UOA p5).

*“I think people in [name of accommodation] do keep to themselves a lot more just because of the way it’s set out”* (FG2 UOA p4).

### Theme 3: The importance of accommodation-based pastoral staff

In university-owned accommodation, residential advisers (RAs) live alongside students in order to provide a source of support:

*“I think having a good RA is important … Having a person there and the RA being visible to the students is really important. In some ways because they are the tie between management and students and also because the RAs are typically of a similar age. It is easier for a student to talk to someone of a similar age than go to an adult especially because they might not want people to know that they are dealing with this issue. It might be a bit easier to just have an informal chat with the RA”* (FG3 UOA p5).

*“I’ve got [name of RA] and she’s so nice. I think it was yesterday or this morning, she came in and just said how are you all getting on kind of thing. She literally just came in for a 10 minute chat like is everything ok? How are you getting on? So I do feel supported by the RA, by her and she also lives next door to me”* (FG2 UOA p5).

However, others highlighted that their RA should be more visible and proactive in getting to know them, as this would enable students to feel comfortable approaching their RA if they have a problem:

*“All the details are there but do you really want to message someone who you’ve met once and be like help me I’m having a crisis I need support?”* (FG4 UOA p1).

*“Even if they just drop by every two weeks for 5 minutes, that would be more than what we’ve had. It would be good for the RA to just come in and say just so you know I’m still here if you want to talk it’s cool”* (FG4 UOA p2).

This is important as often when people feel anxious or depressed, they may withdraw socially and psychologically whilst presenting themselves in a certain way to others with whom they live with. Thus, externally a student may appear fine whilst internally they are struggling, and often this can become exacerbated with students isolating themselves in their bedrooms as they start to feel uncomfortable sharing in terms of communal living:

*“People can present themselves in a certain way and then go back to their room and be depressed, anxious and they don’t want to put on the front as being the depressed person in the flat so they hide it very much because they want to look and fit in with everyone else and not be the person who causes problems by not feeling 100% so I feel like definitely having services share with accommodation is definitely be beneficial and just having little prompts to make sure people are ok can make the world of difference for people”* (FG4 UOA P4).

It would therefore be beneficial for university support services to share information about individual students with accommodation teams to enable them to be in a position to provide additional support to students experiencing mental distress. This is especially important when considering that some students are vulnerable due to pre-existing mental health difficulties, and they may have discontinued the support they were receiving whilst living at home:

*I don’t feel that supported in my accommodation but I haven’t spoken to anyone properly about any struggles I’ve been having or the problems I’ve had. I think it would just be better if, because I know you are down on the university database if you have problems, if the accommodation could reach out because a lot of people do tend to suffer in silence and not want to come forward about things and I know I’ve been like that. I’ve been too scared to go to my kitchen and I’ve literally cried about going to the kitchen because of how anxious it makes me and I just feel like not to be like come and speak to us, just little prompts like we do care, come and speak to us if you do need anything* (FG4 UOA p1).

## Discussion

Attachment to place and relationships with significant others are disrupted by the transition, leaving students vulnerable during this period as they need to re-attach to particular individuals and to a new environment in order to find friends and feel comfortable where they are living. Consistent with previous research (e.g., 17), the importance of forming friends quickly was widely acknowledged. As it is common for young people to arrive at university with the belief that their university experience will be fun-filled with lots of new friends, peer and self-imposed pressure to attend all of the Fresher’s events to bond with their flatmates and cultivate a wider friendship network was keenly felt. Consistent with previous findings (e.g., 20; 21), many young people experienced feelings of loneliness within the first few months, and this was often due to a mismatch between the quantity of relationships that they have and the quantity they expected to have. Friendships between newly acquainted residents living in student accommodation serve an important function as the difficulties students experience during the first few months of university are often unexpected. Some students, however, struggled to form friendships within their flat, and this consequently had a negative impact on their wellbeing. It is common for students to withdraw physically and psychologically when they do not form friendships within their flat. As young people’s support network becomes remote when they move from home to a new university environment, pastoral staff have a very important function to fulfil, especially in supporting those who are most vulnerable.

In line with the elements of biophilic design, our findings highlight aspects of the communal and social areas such as natural light, plants and bright colours, which enable students to feel ‘at home’. Other students, however, described not feeling ‘at home’ in their accommodation. Physical attributes of place can prevent social cohesion as some students found themselves unable to socialise in their living space. Examples of this within flats included the provision of a breakfast bar facing a wall, couches that were unsuitable to accommodate all flatmates, and long corridors of individual rooms that students feel disinclined to go down. Drawing on Thrift’s definition of relational space [12], the areas included in student accommodation are entirely spaces that contain rather than spaces that encourage meaningful relational activity. Accommodation environments need to become places of belonging, which incorporate relational spaces designed with diverse interactions in mind so that a range of group activities can be accommodated and supported. These should be flexible environments where cohesive student communities can grow and thrive.

Although these findings clearly demonstrate the importance of the human element within accommodation, designing places that facilitate community, a sense of we-ness, and belonging is crucial. A number of recommendations are suggested by the findings.

### Recommendations

#### Deliver buildings that promote mental health and wellbeing

Consistent with a recent report on student living [7], accommodation providers should follow the wider architecture sector by building for wellbeing. In particular, accommodation providers should bring human health and wellness to the forefront of building practices and pursue WELL certification. In order to deliver buildings that promote health and wellbeing, future development projects should consider: building in more relational spaces on each floor, organising student bedrooms in clusters rather than linear tranches, ensuring the accommodation is well-lit by natural light, connecting the accommodation to nature, and incorporating bright colours to create a welcoming environment.

A number of physical attributes of place that prevent social cohesion within flats were highlighted. As some students only had a breakfast bar facing a wall, accommodation providers should provide a communal table to promote communal dining within flats. Communal tables add an element of homeliness, and the provision of a table would make young people’s dining experience comfortable. It is also important for students to have comfortable communal areas within flats as the couches were often mentioned as being either too small or too uncomfortable, and this prevented students from socialising together in these areas.

As large social and communal spaces were a student priority, the built environment should facilitate connections by having areas where students can bump into each other and have informal daily conversations. There could be ‘bump spaces’, defined as spaces where you tend to bump into other people rather than having to seek them out, on each floor to enable students to meet and socialise with their neighbours. Ideally kitchen areas should work this way but, as illustrated, these areas were often more cause for concern than areas for informal meetings. There would, however, need to be something to attract students to these ‘bump spaces’ such as nature or art. Similarly, increasing access to community spaces within the built environment such as community gardens and community allotments would be beneficial.

Beginning with an element of homeliness and using biophilic design principles, providers should consider co-designing university accommodation spaces with their student users when it is time to refurbish or to add more provision. They should also consider regular post-occupancy monitoring to see if accommodation meets the needs of students. The co-design of space supports the wellbeing of those involved in the process by enabling students to engage in activities that are themselves co-produced, as well as meeting up for the intrinsic purpose of being with others [25]. It is possible that the co-design of spaces would enhance the student experience as research, conducted in a different setting (e.g., care home), demonstrates that when residents were involved in the design of their collective space, feelings of comfort and social identification were enhanced as rather than simply being ‘in a home’, residents felt ‘at home’ [26].

#### Create and maintain a supportive psychological climate

Consistent with previous research [27], accommodation teams should invest time in organising incoming first year students in order to create and maintain a supportive psychological climate. When students apply for accommodation, they could be given the option to provide information about their personality in order to organise flats according to personality dimensions (e.g., a flat for conscientious students). In addition to this, students could also be given the option to live with similar others such as other international students or those who are undertaking the same course (e.g., flat for arts-based students).

#### Adopt a psychologically informed approach

In order to cater for the emotional and psychological needs of students, accommodation teams should create a psychologically informed environment underpinned by attachment theory [28]. Accommodation staff should invest time into developing relationships or ‘secure attachments’ with students to enable them to thrive and feel psychologically safe within their accommodation environment. For example, pastoral staff could be a ‘secure base’ or ‘safe haven’ for students in order to provide a space for each student to feel protected and supported as this kind of relational or psychologically informed environment fosters agency, connection, meaning and trust which ultimately enables students to flourish.

As students may withdraw physically and psychologically when they do not form friendships within their flat, all pastoral staff should be proactive in supporting students’ wellbeing by providing post-occupancy support. This would involve regularly interacting with students to ensure they are satisfied with their living arrangements. By allowing each student to develop a trusting relationship with pastoral staff, they are more likely to disclose important information and seek support before reaching a point of crisis.

#### Foster a supportive community

Consistent with the Student Mental Health Charter developed by Student Minds [29], the importance of student accommodation as a venue for community building interventions that support social cohesion was highlighted, and examples of group-based activities include creative writing groups, arts-based workshops, shared reading groups, music groups or film-making groups. As many efforts to support social integration and the creation of friendships within university are often ad hoc and unevaluated [29], future research should evaluate programmes that aim to create a sense of community cohesion and reduce feelings of loneliness. As cities tend to support a large number of social enterprises (e.g., ‘The Reader Organisation’) running group-based activities in student accommodation would also connect students to the city and enhance feelings of belongingness.

As students valued the opportunity to talk openly during the focus groups, accommodation teams could organise regular meet-ups with a communal space to talk. This would enable students to share their experiences with similar others in order to provide and receive help. This space may also reduce feelings of loneliness by providing a form of social support. Interest groups could also be formed where students gather to discuss topics, such as sports or politics, amongst themselves. There is even the potential to help in the setting up of study and revision groups where peer support could flourish.

### Limitations

Our findings and recommendations should, however, be considered in light of several limitations. As participants were studying at two universities in northern England, our findings may not be generalizable to the whole student population. Participants in this research were predominantly female, which also limits the generalizability of these findings. As this research was conducted in the first few months of university, students’ views on their living arrangements and friendships may change throughout their first year. Thus, this research only provides a snapshot view of the role of accommodation in student mental health, and it would be beneficial to conduct follow-up focus groups to explore students’ experiences within their accommodation environment over time. Last, as the research was conducted across two institutions with different models of student accommodation, there will have been differences in built form and in ethos between university-owned accommodation and privately owned accommodation. Future research could explore the similarities and differences between university-owned accommodation and privately owned accommodation.

### Conclusions

To conclude, our findings highlight the role of accommodation in social integration and developing friendships. The social ties cultivated through living with compatible others are crucial during this period, especially as students’ primary support networks become remote and their expectations of university often conflict with the reality of the transition. Accommodation providers should design places that facilitate community, a sense of we-ness, and belonging, and accommodation teams should facilitate the formation of friendships and cultivate a sense of community. Taken together, cultivating environments and communities that are supportive of mental health and wellbeing is crucial.

## Supplementary Information

**Additional file 1.**

## Data Availability

Qualitative data extracts are presented in the article to support the findings. The data generated and analysed during the current study are not publicly available as the data collected is sensitive and could compromise the confidentiality and anonymity of the participants but are available from the corresponding author on reasonable request.
