# Supplementary material for: The role of accommodation environments in student mental health and wellbeing
Source: BMC Public Health. 2021 Mar 23;21:573. doi: 10.1186/s12889-021-10602-5 (PMC7986561; doi:10.1186/s12889-021-10602-5)
Supplement: Supplementary file 1 — Additional file 1. [file 12889_2021_10602_MOESM1_ESM.docx]

**The role of accommodation environments in student mental health and wellbeing**

Joanne D. Worsley^1*^, Paula Harrison-Woods^2^, & Rhiannon Corcoran^3^

^1^Department of Psychology, University of Liverpool, Liverpool, UK

^2^Student Administration and Support, University of Liverpool, Liverpool, UK

^3^Department of Primary Care and Mental Health, University of Liverpool, Liverpool, UK

*Correspondence to [jworsley@liverpool.ac.uk](mailto:jworsley@liverpool.ac.uk)

**Focus group topic guide**

**Transition**

***General***

How did you find coming to university?

Are there any factors that affect the mental health of people going to university?

Tell us about any aspects of the university experience that have been stressful.

Can you tell us whether any of the aspects you identified above have affected your mental health or well-being? If so, can you describe in what way.

Tell us what the university could do to make the university environment better or less stressful.

Some students find leaving home and starting university quite difficult, what do you think are the reasons for this?

What was the most difficult part of adapting to university life?

***Schools and colleges***

Do you think there is a need to support people when they move from school or college to university?

Did your school do anything to ease the transition?

Can you think of anything that could be done in schools or colleges to ease the process of going to university?

***Comparisons***

Tell me about your experience of university so far.

When looking at your high school friends’ experiences of university on social media, how does your experience compare?

Do you feel envious of other people’s university experience?

***Sense of belonging/sense of feeling at home***

Do you feel as though you belong to this university? Why?

Do you get a sense of a shared community here? Do you feel ‘at home’ here?

Is student community important? Do you feel part of it?

Did you miss home at all when you came? Do you think that’s important to settling down?

**Accommodation**

***General***

Tell me about your accommodation.

What do you like about your accommodation?

What is not so good about your accommodation?

So the parts that you don’t like, how would you change them?

What factors influenced your decision to choose this hall?

What are the advantages of living on campus/off campus?

Are there any disadvantages of living on campus/off campus?

How safe do you feel in halls? Why?

***Flatmates***

Tell me about meeting your new flatmates / How did you form friendships with your flatmates?

How did you spend the first few days with your flatmates?

How do you spend time with your flatmates nowadays? / How do you socialise with your flatmates nowadays?

Are you happy with your flatmates? / Do you get along with your flatmates?

How did you find ‘fitting in’ in halls?

What are the main benefits of living with other students?

Sometimes when students first come to university they find it difficult to share a space in a co-operative way so they might leave loads of washing up if they are in self-catering or they might want to watch something on TV that others don’t want to watch. What are the difficulties of living with other students? / Are there any problems with living with other students?

Do you engage with other students through cooking?

How can we encourage people to spend more time together in their flats?

***Social and communal areas***

Do you have social and communal areas in your hall?

Are there benefits of having/using social and communal spaces in halls?

Are there disadvantages of not having/using social and communal spaces in halls?

Have you thought of anything else that could be improved? Is there anything else that would help you socialise?

What makes a comfortable space to be in with your friends?

***Allocation***

So how many rooms have you got in your flats? What do you think would be the best number?

Some people think that it might be a good idea if accommodation is organised so that people from the same courses can be together. What do you think about that?

Is there another way that students can be allocated?

***Events and services***

Tell me about your experience of Fresher’s week.

Have you attended any of the small-scale events organised by your Residential Advisor?

Are you aware of your halls student committee?

Are there any services that you feel your accommodation should provide?

Can you think of anything that could help students before they move into halls?

**Expectations**

People often say university should be the best years of your life, what do you think about that?

What expectations did you have when starting university? / What did you expect university to be like? Is it like that? / Have you found that is realistic? / Is that what you’re experiencing now? / Is that what it’s like? What expectations did you have about student accommodation when starting university? Were these expectations met?

What set up those expectations for you? / Why did you have those expectations?

Did social media play a role in setting expectations for you? / Did social media have anything to do with how you set your expectations up?

Are you finding that coming to university meets your expectations?

Overall, how would you rate your university experience [worse than expected, as expected, better than expected] and why?

So if it is not as good as you expected it to be, does that affect your well-being or how you feel about life?

**Friendships/groups**

Some people go through university without making friends or joining any clubs or societies. Describe what you think it would be like if you did not have a strong friendship group or tell us what it is like if you do not have a strong friendship group.

Tell us about making friends at university (e.g., in halls, on your course, or joining clubs and societies).

What helped you to make friends at university?

Is there anything that could have helped you to make friends easier?

Do you feel that you have enough university friends? What has helped you or what has stopped you?

Some people like to have a few close and other people like to have lots of looser friendships, which do you think is better to support you through university?

Is having friends or belonging to different social groups important to the university experience? If yes, why? If no, why not?

Since starting university, have you joined any societies or social groups? Tell us what they are and what it has been like.

Do you think it’s valuable to have a student union? / How important is the student union in making friends?
